# Synthesis and Characterization of LiFePO_4_–PANI Hybrid Material as Cathode for Lithium-Ion Batteries

**DOI:** 10.3390/ma13122834

**Published:** 2020-06-24

**Authors:** Cesario Ajpi, Naviana Leiva, Max Vargas, Anders Lundblad, Göran Lindbergh, Saul Cabrera

**Affiliations:** 1 Department of Inorganic Chemistry and Materials Science/Advanced Materials, IIQ Chemical Research Institute, UMSA Universidad Mayor de San Andres, La Paz 303, Bolivia; nindeleivqmc@gmail.com (N.L.); vargmax@gmail.com (M.V.); 2 Department of Chemical Engineering, Applied Electrochemistry, KTH Royal Institute of Technology, SE-10044 Stockholm, Sweden; gnli@kth.se; 3 Division of Safety and Transport/Electronics, RISE, Research Institutes of, Sweden, SE-50462 Borås, Sweden; anders.lundblad@ri.se

**Keywords:** PANI, LiFePO_4_, conducting polymers, hybrid materials, lithium-ion batteries

## Abstract

This work focuses on the synthesis of LiFePO_4_–PANI hybrid materials and studies their electrochemical properties (capacity, cyclability and rate capability) for use in lithium ion batteries. PANI synthesis and optimization was carried out by chemical oxidation (self-assembly process), using ammonium persulfate (APS) and H_3_PO_4_, obtaining a material with a high degree of crystallinity. For the synthesis of the LiFePO_4_–PANI hybrid, a thermal treatment of LiFePO_4_ particles was carried out in a furnace with polyaniline (PANI) and lithium acetate (AcOLi)-coated particles, using Ar/H_2_ atmosphere. The pristine and synthetized powders were characterized by XRD, SEM, IR and TGA. The electrochemical characterizations were carried out by using CV, EIS and galvanostatic methods, obtaining a capacity of 95 mAhg^−1^ for PANI, 120 mAhg^−1^ for LiFePO_4_ and 145 mAhg^−1^ for LiFePO_4_–PANI, at a charge/discharge rate of 0.1 C. At a charge/discharge rate of 2 C, the capacities were 70 mAhg^−1^ for LiFePO_4_ and 100 mAhg^−1^ for LiFePO_4_–PANI, showing that the PANI also had a favorable effect on the rate capability.

## 1. Introduction

Since Sony developed and commercialized the first lithium-ion batteries based on LiCoO_2_ in 1990, new materials and different applications have driven research and development. One direction has been in the development of materials with improved electrochemical properties (specific energy, energy density, rate capability and cyclability) for hybrid and electric vehicles and energy storage systems. Materials that have been extensively studied include inorganic materials such as LiFePO_4_ [1,2] and LiMn_2_O_4_ [3,4]; organic materials such as Li_4_C_6_O_6_, quinones and anthraquinones [5]; polyaniline (PANI); and others.

LiFePO_4_ has two main disadvantages: low electronic conductivity and slow Li-ion diffusion due to its 1D channel for Li extraction. Efforts have been made to improve the electrochemical performance by carbon coating [6], cation doping [7,8] or minimizing the particle size [9,10]. Carbon coating is a common method to enhance the electronic conductivity of LiFePO_4_. The carbon is electrochemically inactive, but its incorporation in a LiFePO_4_ electrode can influence the capacity and cyclability. Substituting the inactive carbon black and binder typically present in composite electrodes with an electrochemically active polymer like polypyrrole (PPy) or polyaniline (PANI) has been proposed [11,12,13,14,15,16,17], to enhance the electrochemical performance. PANI is a promising conducting polymer due to its facile synthesis, environmental stability and tunable physical and electrochemical properties controlled by oxidation and protonation [18]. PANI is electrochemically active in the range of 2.0−3.8 V, which overlaps with the redox couple of LiFePO_4_. Hybrid electrodes for lithium-ion batteries incorporating PANI with LiMn_2_O_4_ [19], MnO_2_ [20], V_2_O_5_ [21] and Li(Mn_1/3_Ni_1/3_Fe_1/3_)O_2_ [22] have been reported. PANI shows compatible polarity with the electrolyte via the formation of H bonds. PANI can mediate the polarity difference between the cathode particles and the electrolyte, promoting electrolyte permeation into the surface of the active particles and hence enhancing Li-ion extraction/insertion during a charge/discharge process. Incorporating LiFePO_4_ with conductive PANI is thereby an attractive route to improve both electronic conductivity and Li-ion diffusion. The capacity of PANI is usually higher than that of PPy or PT, but depends on doping [23]. Various dopants have been used to improve the physical and chemical properties of PANI. Among them, salts such as LiClO_4_, LiBF_4_, LiPF_6_ and Zn(ClO_4_)_2_ have received much attention, and their application in rechargeable lithium-ion batteries has been extensively studied [24].

In general, PANI and LiFePO_4_–PANI are synthesized via oxidative polymerization in acidic media. For the synthesis of PANI, various oxidants, such as ammonium peroxydisulfate (APS) [25], hydrogen peroxide [26], ferric chloride [18] and ferric sulfate [27], have been used. With APS as the oxidant, PANI can be successfully doped with inorganic acid (e.g., HCl, H_2_SO_4_ and H_3_PO_4_) [28] or organic acid (e.g., dicarboxylic acid) [18]. The most common method for the synthesis of LiFePO_4_–PANI is the shelf-assembly process, PANI is incorporated with LiFePO_4_ particles through simultaneous chemical polymerization with APS as the oxidizer, and aniline and an inorganic acid as dopants [12].

The overall chemical and electrochemical properties can be improved with advanced hybrid materials combining inorganic and organic materials. For the design of this type of hybrid material, the interactions between the inorganic and organic parts are key (supramolecular chemistry); this opens up an immense number of possibilities for different combinations [29].

This work is focused on the synthesis, structural characterization and electrochemical characterization of LiFePO_4_–PANI as a cathode material for lithium-ion batteries. PANI was prepared via a self-assembly process, commercial LiFePO_4_ was used and a LiFePO_4_–PANI hybrid material was synthetized by a solid-state reaction. The main challenge in the synthesis of LiFePO_4_–PANI via self-assembly process is LiFePO_4_ dissolution by the acid (e.g., HCl, H_2_SO_4_ and H_3_PO_4_) and oxidation of the LiFePO_4_ to FePO_4_ by APS, resulting in low reaction yields. The method used for the synthesis of LiFePO_4_–PANI in this study does not use APS and acids, which is advantageous in terms of reaction yield. PANI and AcOLi were simultaneously incorporated with LiFePO_4_ particles through a thermal treatment. Additionally, the method incorporates Li-ions in the PANI structure as dopants. XRD, FTIR and TG were used for the characterization of LiFePO_4_, PANI and LiFePO_4_–PANI, and the surface morphology was studied by using SEM. The electrochemical behavior and discharge/charge performance were investigated by EIS, CV and charge/discharge processes. Rate capability and cyclability were measured for the LiFePO_4_–PANI and showed improvement when compared to the pure materials.

## 2. Materials and Methods

### 2.1. Materials Synthesis of PANI

First, 25 mmol of ammonium persulfate (APS) (98%, Sigma-Aldrich, St. Louis, MS, USA) was dissolved in 50 mL of distilled water. Then, 20 mmol of aniline was dissolved in 50 mL of 1M of H_3_PO_4_ (ACS reagent, ≥85 wt%, Sigma-Aldrich, St. Louis, MS, USA) in aqueous media. Both solutions were prepared at room temperature. The APS solution was added to the solution containing the aniline. The mixture was magnetically stirred for 1 h, at 5 °C. The dark blue precipitate of emeraldine base (EB) was collected by filtration and washed 5 times with distilled water and 5 times with ethanol and dried under vacuum, at 60 °C, for 8 h.

### 2.2. Synthesis of LiFePO_4_–PANI

The synthesis of LiFePO_4_–PANI with ~25% polyaniline was carried out by using 0.75 g of commercial LiFePO_4_ (Phostech Lithium Inc., St-Bruno-de-Montarville, Quebec, Canada, coated with 2% of C), 0.25 g of synthetized PANI (i.e., EB) and 0.1675 g of lithium acetate (AcOLi) (99.95%, Sigma-Aldrich, St. Louis, MS, USA). The amounts of the compounds were mixed in a mortar for 0.5 h. After that, the mixture was transferred to a crucible and thermally treated at 300 °C, for 1 h, in an Ar/H_2_ (90/10) atmosphere. A black powder was obtained with a weight yield of 99.98%.

### 2.3. Structural Characterization

The LiFePO_4_–PANI was characterized by X-ray powder diffraction (PXRD), using an X’Pert3 PANalytical diffractometer with a scan rate of 0.02 °/s, by Cu–Kα radiation (λ = 1.5406 Å, 45 kV, 20 mA). X’pert Highscore software (PANalytical. B. V, Lelyweg, the Netherlands) was used for the identification of the phases. The morphologies of the samples were determined by S-4800 series field-emission high-resolution scanning electron microscope (SEM) (Hitachi, Tokyo, Japan), equipped with an EDS detector (Oxford Instruments, Abingdon, UK). The powder samples were mounted in the sample holder and sputtered with a thin layer of Pt/Pd. Fourier transform infrared spectroscopy (FTIR) was done on a Spectrum 100 spectrometer (Perkin Elmer), in the wavelength range of 4000–400 cm^−1^, with a resolution of 1 cm^−1^. Thermogravimetric analysis (TGA) (LABSYS evo STA 1150, SETARAM Instrumentation, Caluire-et-Cuire, France) was carried out on 20 mg of specimen, from room temperature to 800 °C, with a heating rate of 2 °C/min.

### 2.4. Electrode Preparation and Electrochemical Characterization

The active cathode material was made by preparing an 80:10:10 mixture of active material (LFP–PANI), conducting agent (Super P) and binder (PVDF), respectively. The components of the mixture were weighed and subsequently ground for 15 min. Then N-methylpyrrolidone (NMP) was added as solvent, and the mixture was magnetically stirred for 1 h. The electrodes were tape-casted by a doctor blade process on an Al foil with a slit height of 100 µm. The electrochemical testing was done in a bottom-type test cell of 0.9 cm^2^ active area, using metallic Li as the counter and reference electrode, Celgard (2325) as separator and a solution of 1 M LiPF_6_ dissolved in EC-DMC (1:1 vol) as electrolyte. The test cells were subjected to charge–discharge processes at a rate of 0.1 C between 2.5 and 4.2 V vs. Li^+^/Li^0^ and electrochemical impedance spectroscopy (EIS) measurement in the frequency range between 100 kHz and 0.1 Hz, with an AC voltage amplitude of 10 mV, using a Gamry potentiostat 600.

## 3. Results and Discussion

### 3.1. Synthesis of PANI and LiFePO_4_–PANI

**Figure 1:**
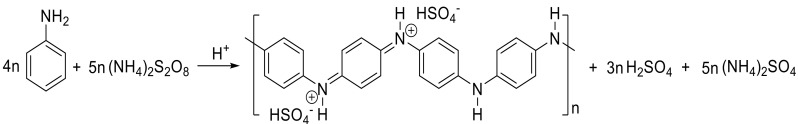
Reaction for the synthesis of PANI.

The synthesis of PANI was performed by chemical oxidation of aniline with APS; the reaction is shown in Figure 1.

**Figure 2:**
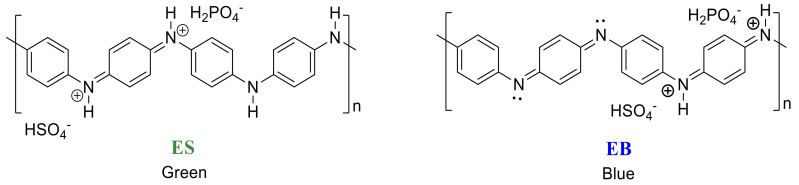
Structure of ES (emeraldine salt) and EB (emeraldine base).

The addition of aniline to the solution of H_3_PO_4_ leads to the formation of protonated C_6_H_5_NH_3_^+^ -H_2_PO_4_^−^, which is more soluble in water compared to aniline and reacts with the APS, initiating the polymerization. A possible reaction mechanism for this is shown in Supplementary Materials Figure S1. After the addition of the APS solution, the onset of the polymerization reaction was marked by a green coloration due to the formation of a chemical species of the polyaniline called ES (emeraldine salt). Later, the color changed to blue due to the formation of the chemical species EB (emeraldine base); this change was controlled by the H^+^ concentration (pH). The structures of these species are shown in Figure 2.

**Figure 3:**
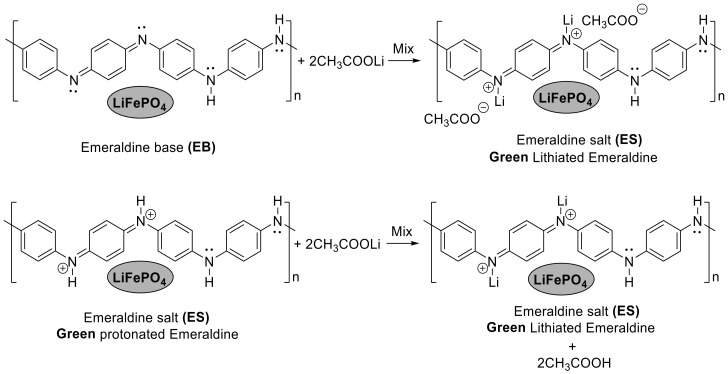
Reactions of ES (emeraldine salt) and EB (emeraldine base) with AcOLi.

The reaction between LiFePO_4_, PANI and AcOLi starts during mixing, noted by a characteristic smell of acetic acid and a change in the color of the PANI from blue to green. The observed change of color was attributed to the reaction in Figure 3.

EB and protonated ES in the PANI structure can be lithiated by reaction with AcOLi, forming (in both cases) lithiated ES. During thermal treatment at 300 °C for 1 h in an inert atmosphere of Ar/H_2_, the AcOH is removed by evaporation.

**Figure 4:**
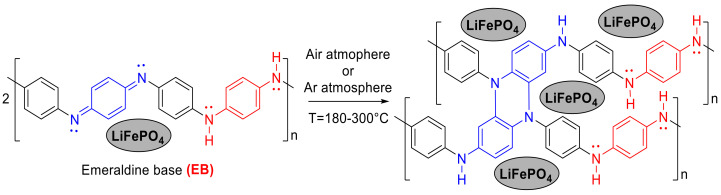
Conversion reaction and structure of crosslinked PANI. A quinoid unit of the PANI chain, shown in blue, reacts with a quinoid unit of another PANI chain, to form crosslinked PANI.

The synthesis of the LiFePO_4_–PANI was carried out at 300 °C. During the thermal treatment, the PANI was converted by a chemical reaction between two chains of PANI [15], as shown in Figure 4.

### 3.2. PXRD Characterizations of LiFePO_4_, PANI and LiFePO_4_–PANI

**Figure 5:**
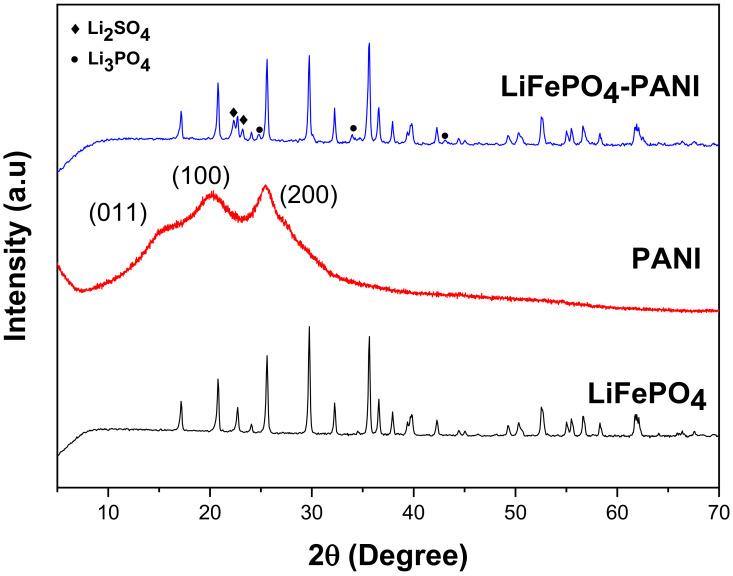
Diffraction patterns of the LiFePO_4_, PANI and LiFePO_4_–PANI samples.

The dark blue PANI powder was characterized by PXRD. The diffraction patterns of the product and the starting powders are shown in Figure 5.

**Figure 6:**
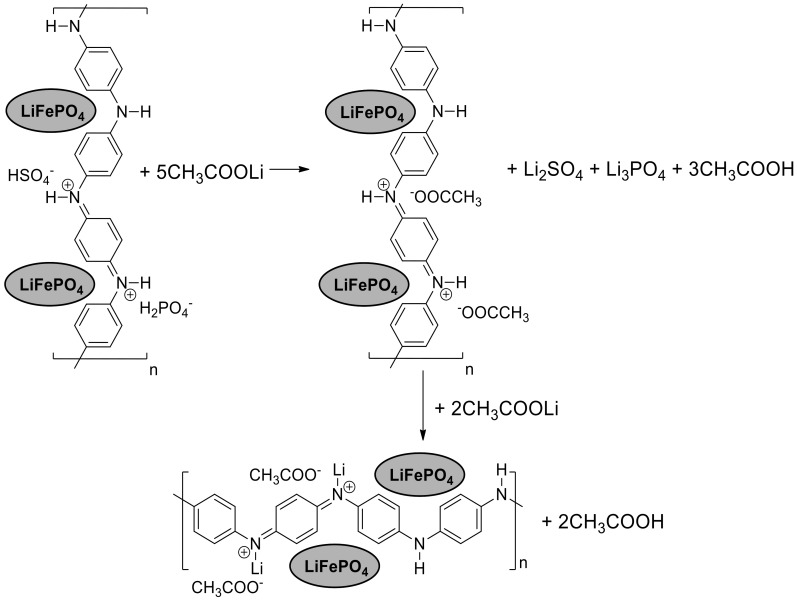
Reactions of the HSO_4_^−^ and H_2_PO_4_^−^ groups in the PANI with AcOLi.

The diffraction pattern shows a higher crystallinity of the pure PANI than normally reported. In general, the crystallinity of PANI depends of the methods of synthesis and dopants [30,31]. The diffraction planes (011) correspond to the orientation parallel to the polymer chain. The planes (100) correspond to the parallel and perpendicular periodicity of the polymer chain. The planes (200) correspond to the periodicity perpendicular along the chain [11]. The XRD diffraction pattern of the LiFePO_4_–PANI composite shows diffraction peaks corresponding to group Pnmb for LiFePO_4_. A low amount of phase impurity is detected as Li_2_SO_4_ (01-075-0929) and Li_3_PO_4_ (01-071-1528). These impurities originate from the reaction between Li^+^ from the AcOLi and HSO_4_^−^ and H_2_PO_4_^−^ in the structure of PANI. A proposed reaction for the formation of Li_2_SO_4_ and Li_3_PO_4_ is shown in Figure 6. The diffraction pattern of PANI in the composite material is not visible, due the lower relative intensity of its diffraction peaks compared to LiFePO_4_. This decreased intensity is due to the small amount and possibly also lesser crystallinity of the PANI deposited on the particles.

### 3.3. Morphologies of LiFePO_4_, PANI and LiFePO_4_–PANI

**Figure 7:**
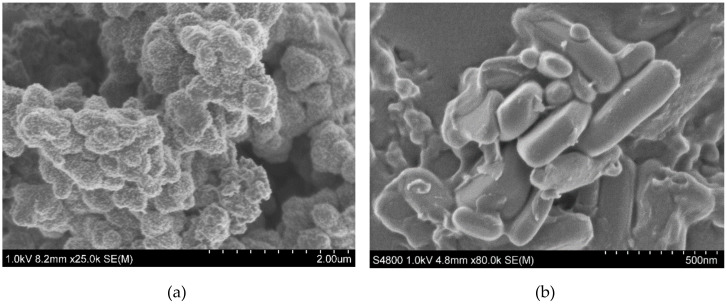

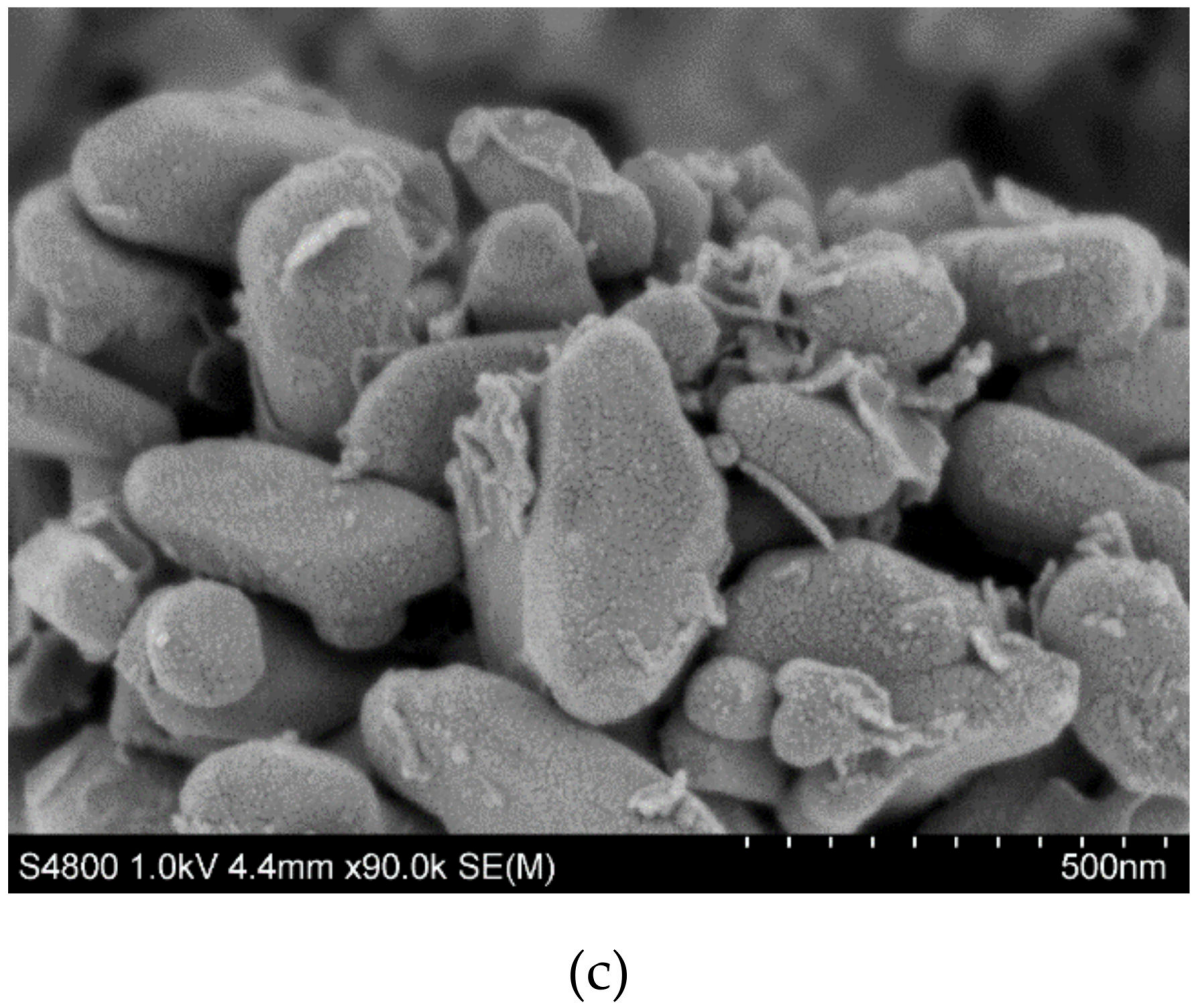
SEM images for (**a**) synthesized PANI, showing the agglomerates; (**b**) LiFePO_4_, showing the particles; and (**c**) LiFePO_4_–PANI hybrid.

Figure 7a–c shows SEM images of PANI, LFP and LFP–PANI, respectively. The resulting PANI powder prepared by a self-assembly process has globular morphology, with aggregates having an average diameter of 2.75 µm, estimated from Supplementary Materials Figure S2. The primary particles have an average diameter of 310 nm and a globular morphology, which are characteristic of PANI synthesized by chemical oxidation, as shown in Figure 7a and Supplementary Materials Figure S2. In Figure 7b, a distribution of LFP particle size can be seen with an estimated average diameter of 300 nm. The LFP–PANI particles have an estimated average diameter of 180 nm, as seen in Figure 7c. The particles were split via milling and stirring in a mortar before the thermal treatment and were homogeneously coated by PANI. This is an advantage for the diffusion of electrolyte and Li^+^ into the active particles and leads to a good rate capability of the material [12].

**Figure 8:**
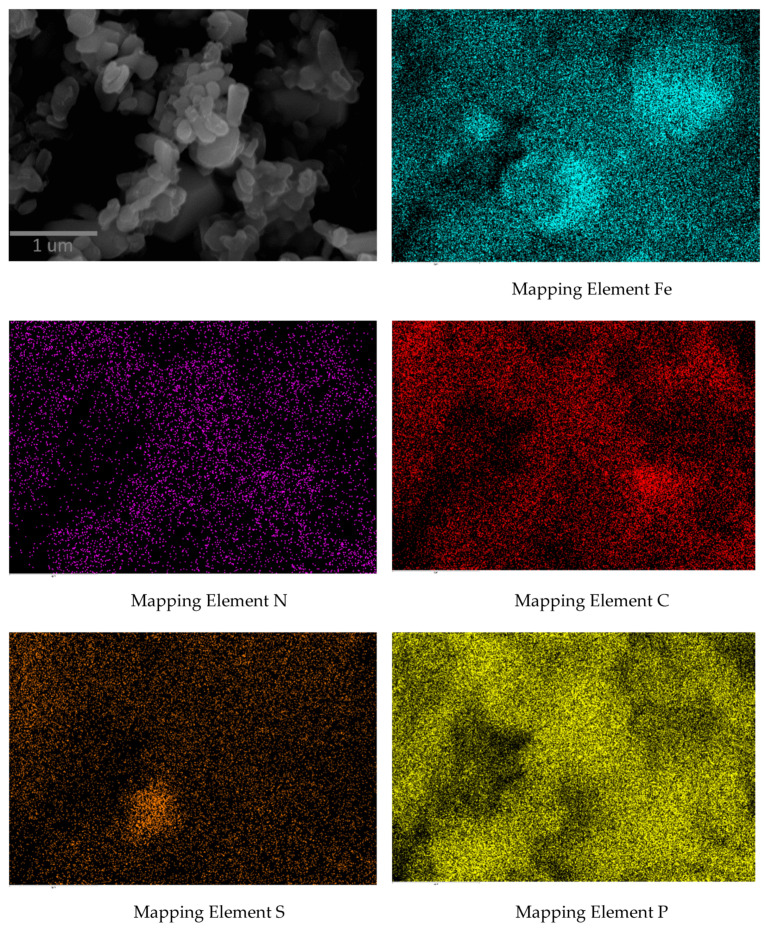
Scanning electron microscopy (SEM) of LiFePO_4_–PANI coupled with energy dispersive spectroscopy (EDS).

The particle size and shape of the LiFePO_4_–PANI composite particles are similar to LiFePO_4_ particles, but the surfaces morphologies are different. Figure 7c shows the expected coating on the composite LiFePO_4_ particles. Scanning electron microscopy (SEM) imaging coupled with EDS mapping provides a qualitative and semiquantitative elemental analysis of the LiFePO_4_–PANI powder and is presented in Figure 8.

**Figure 9:**
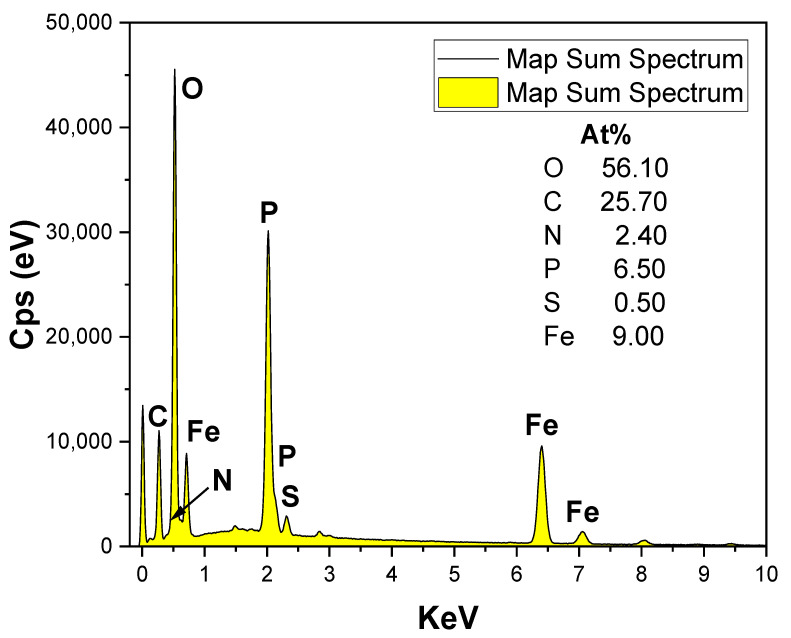
Energy dispersive microscopy (EDS) spectrum for LiFePO_4_–PANI powder.

There is good correlation between the PXRD and the experimental EDS (Figure 9), showing the presence of C, N, P, S and Fe. The maps of N, S and C indicate homogenous distribution of PANI on the LiFePO_4_ particles. However, some flakes of PANI extending from the particle surfaces can also be observed. The mapping of S shows a low concentration attributed to a small amount of Li_2_SO_4_. The EDS spectrum of the synthesized PANI is shown in Supplementary Materials Figure S3.

### 3.4. FT-Infrared Spectroscopy

**Figure 10:**
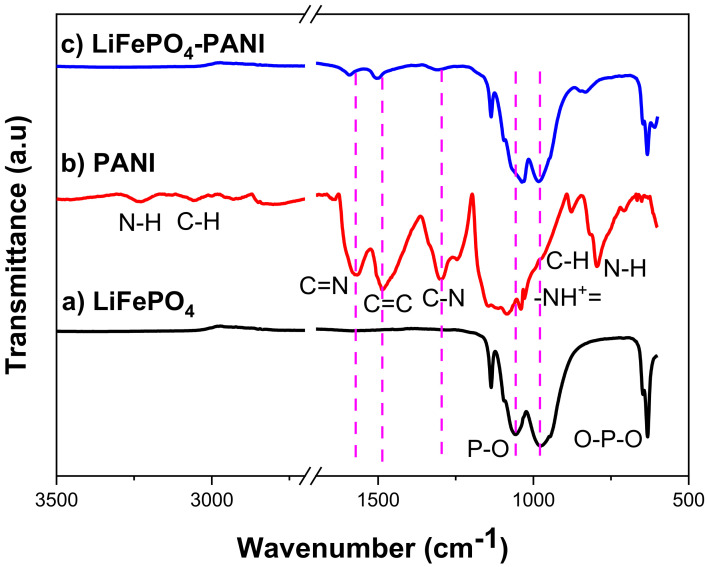
FTIR spectra for: (**a**) LiFePO_4_, (**b**) PANI and (**c**) LiFePO_4_–PANI.

The IR spectrum of LiFePO_4_ in Figure 10 shows four fundamental modes; the bands around 1136, 1092, 1058 and 977 cm^−1^ correspond to the P–O antisymmetric stretching vibration of the olivine phosphate groups [32,33]. The bands around 647 and 632 cm^−1^ correspond to the O–P–O symmetric and anti-symmetric stretching vibrations [34]. The IR spectrum of PANI (Supplementary Materials Figure S4) shows the presence of a broad band around 1050 cm^−1^, which is known as an “electronic band” being associated with the doped form of polyaniline, specifically the vibration mode of the -NH^+^= structures [35]. 

**Figure 11:**
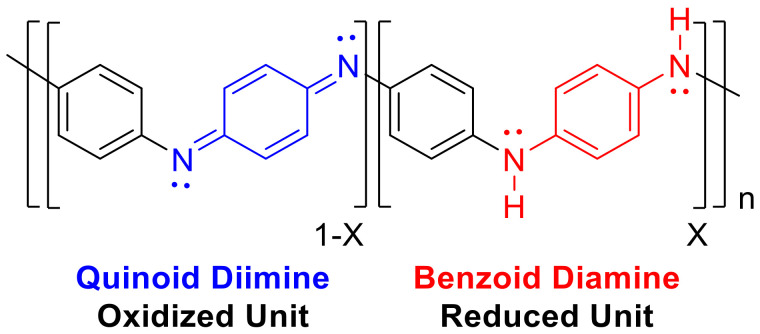
Oxidized and reduced units.

The spectra also show characteristic peaks at about 1575 cm^−1^ for C = N stretching mode of the quinonoid rings (quinoid unit, Figure 11), 1486 cm^−1^ for C = C stretching mode of benzenoid rings (benzoid unit, Figure 10) and 1299 cm^−1^ for C-N stretching mode. The vibrational bands at about 1144 and 708 cm^−1^ are assigned to the aromatic ring in-plane and out-of-plane C-H bending [36,37]. The peak at 796 cm^−1^ is assigned to N-H wag vibration, characteristic of primary and secondary amines only. The peak at 3229 cm^−1^ is assigned to N-H stretching vibration, which is present in the case of PANI-EB. The structure of PANI is generally represented by the following structure, where X = 0.5 for the polyemeraldine salt:

The IR spectrum of LiFePO_4_–PANI shows peak shifts in the broad bands (LiFePO_4_ band to LiFePO_4_–PANI band); 1092 to 1194, 1058 to 1031 and 977 to 983 cm^−1^ correspond to the P–O antisymmetric stretching vibration compared to LiFePO_4_. This is probably due to hydrogen bonding (P-O---H-N) between LiFePO_4_ and PANI. The O–P–O symmetric and anti-symmetric stretching vibrations at 632 cm^−1^ and the band at 647 cm^1^ related to P vibration remain unchanged. A new peak appears at 610 cm^−1^, attributed to the vibration of O---H in the interaction O–P–O---H-N.

The vibration mode at 1136 cm^−1^ associated with -NH^+^= structures of polyaniline is overlapped with the P-O vibration. The peak changes compared to polyaniline from 1299 to 1307 cm^−1^ for the C-N stretching, 1575 to 1592 cm^−1^ for the C = N stretching mode of the quinonoid rings and 1486 to 1505 cm^−1^ for the C = C stretching mode of benzenoid rings reveal that the polyaniline in the LiFePO_4_–PANI has more quinoid units than the PANI. This is probably because the chain of PANI deposited on the surface of the particles of LiFePO_4_ has a greater conjugation.

The peaks at 796 cm^−1^ assigned to N-H wag vibration and at 3229 cm^−1^ assigned to N-H stretching vibration are not present. This is attributed to chemical interaction by the hydrogen bond N-H--O-P. The changes in the vibration values in the LiFePO_4_–PANI compared with LiFePO_4_ and PANI separately are attributed to chemical interaction by hydrogen bonds between the electronic clouds of PANI and LiFePO_4_, which produce polarization and change in the dipolar moment.

The R value was calculated in order to find the ratio of the quinoid and benzenoid units (C = N/C-N). The ratio is directly related to the oxidation state of the PANI polymer. The value can be obtained by taking the ratio of the areas of the IR bands at ~1300 and ~1590 cm^−1^ in Figure 11. The ratio R is calculated on the basis of Equation (1) [37]:

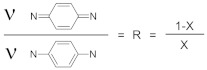
(1)

**Figure 12:**
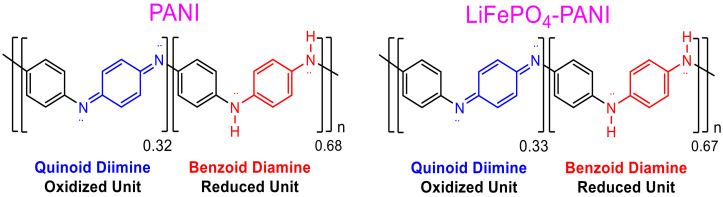
Oxidized and reduced units of PANI and LiFePO_4_–PANI.

The values based on the areas determined from the FTIR data are R = 0.462 for PANI and R = 0.500 for LiFePO_4_–PANI, respectively. When the ratio of the areas of the two bands is less than one, this indicates that there are more benzoid than quinoid units within the PANI polymer. Therefore, the structure of PANI and LiFePO_4_–PANI doped with AcOLi can be represented as shown in Figure 12.

**Figure 13:**
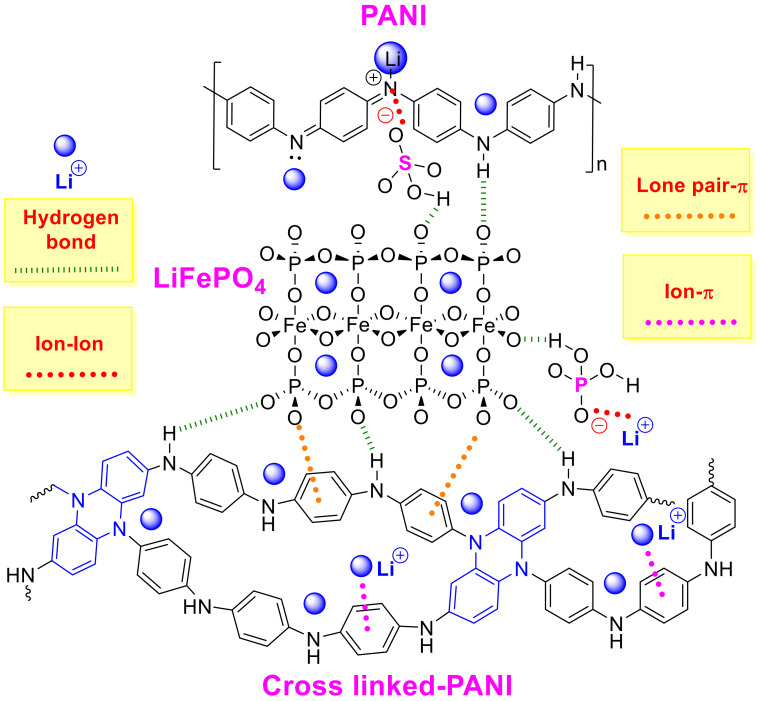
Possible structure of the LiFePO_4_–PANI and its interactions.

The ratio between quinoid and benzoid units in the PANI and LiFePO_4_–PANI are similar. This indicates that the thermal treatment does not produce significant changes in the structure of the PANI. A possible structure and the interaction between the LiFePO_4_ and PANI are shown in Figure 13.

Possible interactions present in the structure are hydrogen bonds, ion–ion, oxygen lone pair–π and ion–π. These interactions are weak and indicate the possibility of interaction and formation of a small amount of crosslinked PANI during the thermal treatment. A possible structure for PANI is shown in Supplementary Materials Figure S5.

### 3.5. Thermogravimetric Analysis

**Figure 14:**
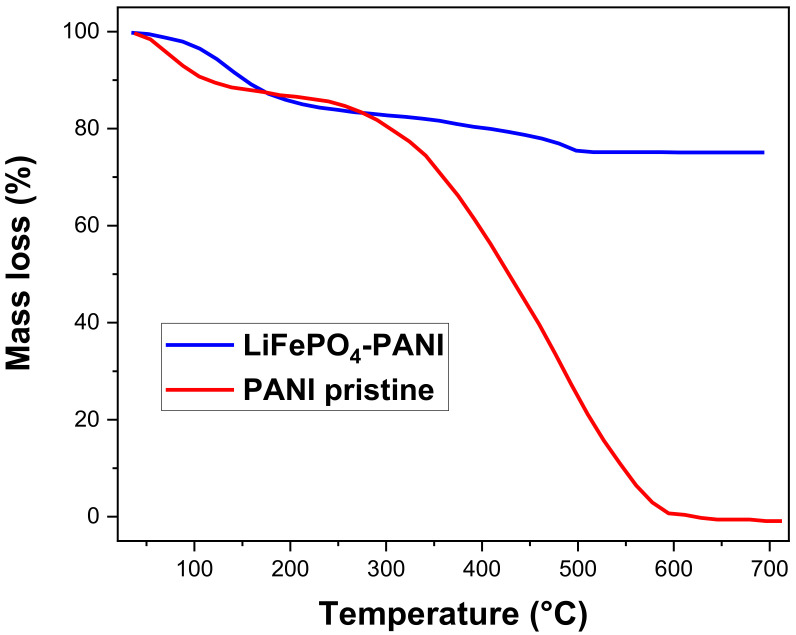
Thermogravimetric curves for pristine PANI and LiFePO_4_–PANI.

The TGA measurements of the PANI and LiFePO_4_–PANI powders were carried out in air atmosphere, and the TGA curves are shown in Figure 14. The mass loss from room temperature to 150 °C is due to the elimination of small amounts of water absorbed in the LiFePO_4_–PANI and PANI. The pristine PANI has a greater mass loss in this temperature interval and, thus, higher water content than the hybrid powder. This can be explained by the heat treatment at 300 °C in inert atmosphere during the synthesis, which dried the hybrid powder. The pristine PANI is completely decomposed between 150 and 600 °C. The PANI in LiFePO_4_–PANI is decomposed at 500 °C. LiFePO_4_ is still stable up to 850 °C, and the residual mass of carbon formed by decomposition of the polymer can be ignored. Thus, the mass content of the polymer can be calculated from the mass loss of the composite in the TGA curve. This calculated mass content of PANI in the LiFePO_4_–PANI composites is 25%, which corresponds well to the precursor weights in the synthesis step. Possible decomposition reactions are shown in Supplementary Materials Figures S6 and S7.

### 3.6. Electrochemical Characterization

**Figure 15:**
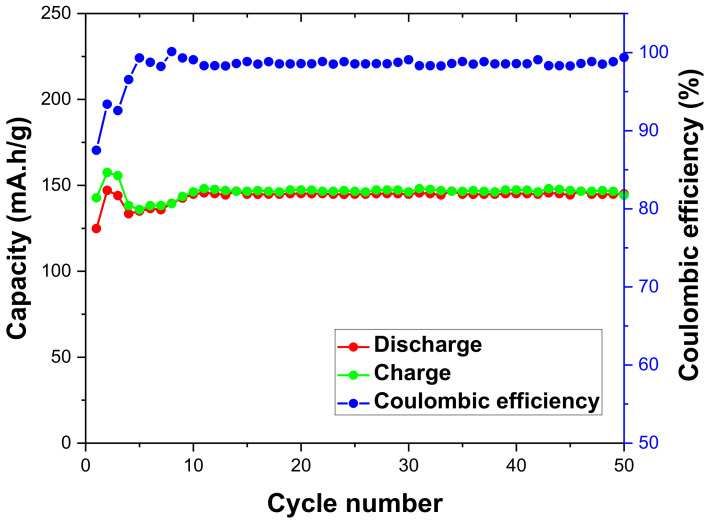
Charge/discharge cycling performances for the LiFePO_4_–PANI composites. The data were obtained by charging and discharging at 0.1 C.

The LiFePO_4_–PANI composites were electrochemically tested in a button-type test cell by charging and discharging at room temperature over 50 cycles, at a rate of 0.1 C. Figure 15 shows the charge–discharge capacity vs. cycle number for the LiFePO_4_–PANI composite. The LiFePO_4_ and LiFePO_4_–PANI doped with Li^+^ are electrochemically active in the range of 2.4–4.2 V. The LiFePO_4_–PANI shows a small capacity fade of about 0.2% over 50 cycles.

**Figure 16:**
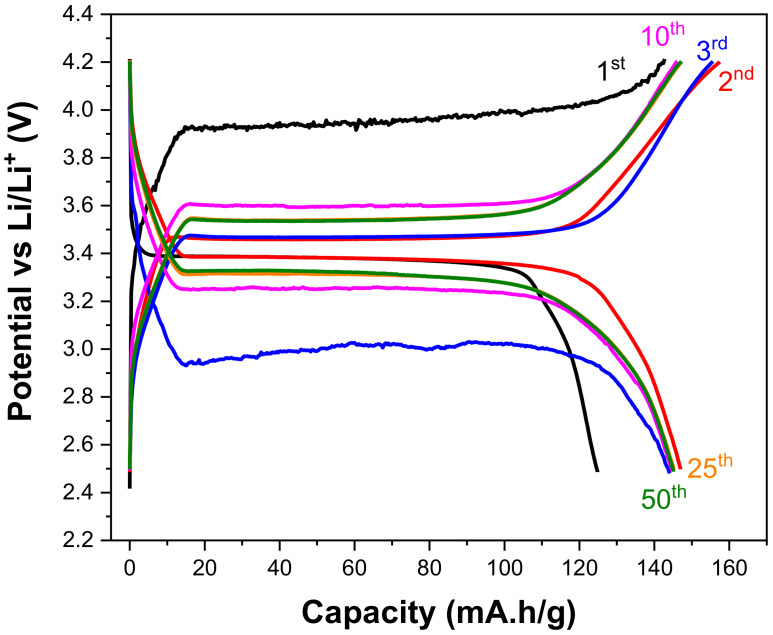
Cell voltage as a function of specific capacity obtained by charging and discharging at the same rate for the LiFePO_4_–PANI. The data were obtained by charging and discharging at 0.1 C.

The cell voltage as a function of specific capacity for LiFePO_4_–PANI composites tested at 2.4–4.2 V is shown in Figure 16. The inserted number of Li-ions determines the specific capacity of the materials, and the occupation sites of Li-ions control the electrochemical potential. LiFePO_4_–PANI materials have one equivalent site in LiFePO_4_, which suggests the same site energy for each Li-ion, resulting in a single continuous discharge plateau and a constant cell voltage. Supplementary Materials Figure S10 shows the charge and discharge profiles at 0.1 C as a function of time.

**Figure 17:**
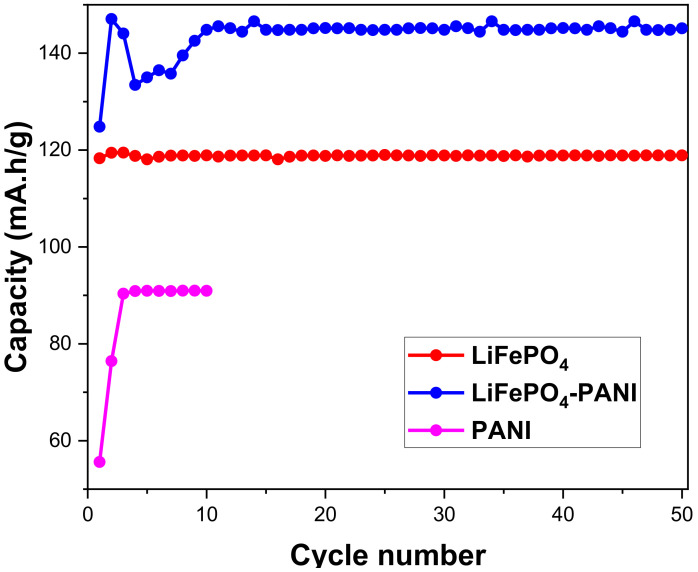
Comparison of discharge cycling performances for the LiFePO_4_–PANI composite and LiFePO_4_. The data were obtained by charging and discharging at 0.1 C.

From Figure 17, the specific discharge capacity of LiFePO_4_–PANI at 0.1 C is 145 mAh g^−1^, and the corresponding result for pure LiFePO_4_ is 120 mAh g^−1^. The theoretical capacity of PANI is 142 mAh g^1^, and that of LiFePO_4_ is 170 mAh g^−1^. The coulombic efficiency of the LiFePO_4_–PANI reaches 98%. The LiFePO_4_–PANI composite with 25 wt.% PANI exhibits a superior performance with a specific capacity of 145 mAh g^−1^, compared to 120 mAh g^−1^ for pure LiFePO_4_ and 95 mAh g^−1^ for pure PANI, as shown in Figure 17. This can be, in part, ascribed to the improved electrical conductivity of LiFePO_4_ by its interaction with the PANI structure. The charge–discharge and cell voltage curves of the PANI are shown in Supplementary Materials Figures S8 and S9.

**Figure 18:**
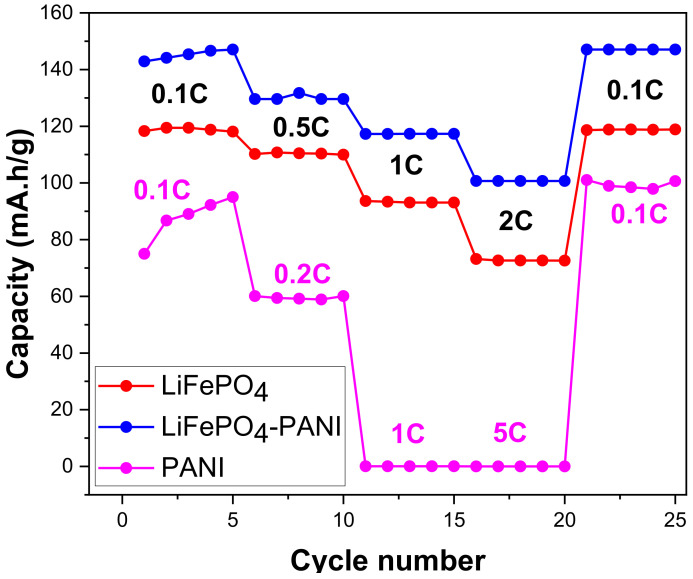
Specific discharge capacity at different C-rates for the LiFePO_4_–PANI, PANI and pure LiFePO_4_. The cells were charged and discharged at the same rates and conditions, from 0.1 C to 2 C.

PANI, LiFePO_4_ and LiFePO_4_–PANI cycled at different C-rates are compared in Figure 18. The LiFePO_4_–PANI shows the best rate capability with discharge capacities of 145 mAh g^−1^ at 0.1 C and 100 mAh g^1^ at 2 C. This means that incorporation of PANI on the surface of the particles of LiFePO_4_ results in 21% capacity enhancement at 0.1 C and 45% enhancement at 2 C. The enhanced rate capability is attributed to the improved electrical and ionic conductivity and Li^+^ diffusion promoted by PANI at the surface of the LiFePO_4_ particles.

**Figure 19:**
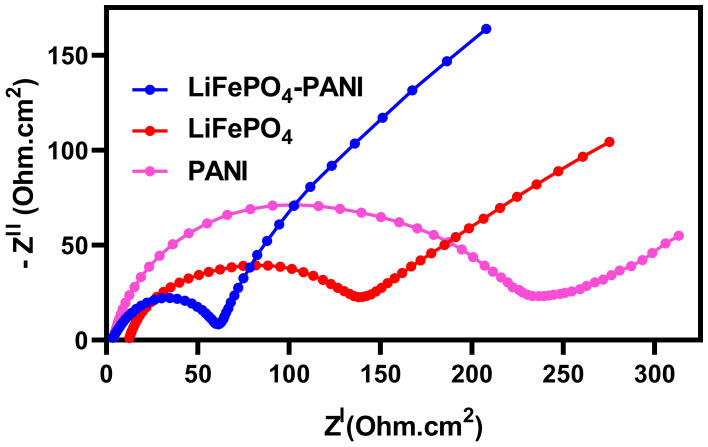
Experimental Nyquist curves for PANI, LiFePO_4_ and LiFePO_4_–PANI.

The electrochemical impedance spectroscopy (EIS) curves for the PANI, LiFePO_4_ and LiFePO_4_–PANI composite cathodes at OCV (2.40 V for PANI, 2.17 V for LiFePO_4_ and 3.42 V for LiFePO_4_–PANI) are shown in Figure 19.

The high frequency real axis-intercept, R_1_ in Table 1, represents the ionic bulk resistance and the electrical contact resistance, and the semicircle formed from the middle frequency range of the impedance spectra represents the charge transfer resistance, R_ct_ [38,39]. The smaller diameter of the LiFePO_4_–PANI semicircle shows a lower charge transfer resistance (R_ct_) for the composite electrode, as shown in Table 1. This indicates that the LiFePO_4_ coated or combined with conductive PANI can effectively improve the charge transfer and the electrochemical properties of LiFePO_4_ particles.

**Figure 20:**
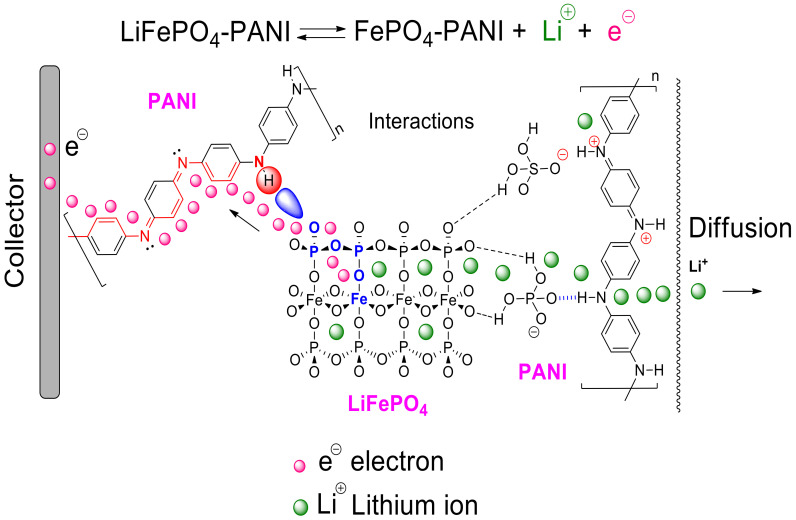
Scheme of the transfer and electron and diffusion of Li^+^.

The results can be correlated with Figure 20, which shows the different processes in the proposed structure. The PANI also reduces the contact resistance by increasing the contact area between LiFePO_4_ particles and the current collector. Figure 20 shows the electrochemical reaction of LiFePO_4_ and the scheme of the LiFePO_4_–PANI charging process. In the charge transfer process, the electron first moves through the molecular orbital network in the LiFePO_4_. After that, the electron is transferred to the PANI chain and moves through the polymer chain to the current collector. The Li^+^ moves through the network of the LiFePO_4_ structure and the PANI structure to the electrolyte.

**Figure 21:**
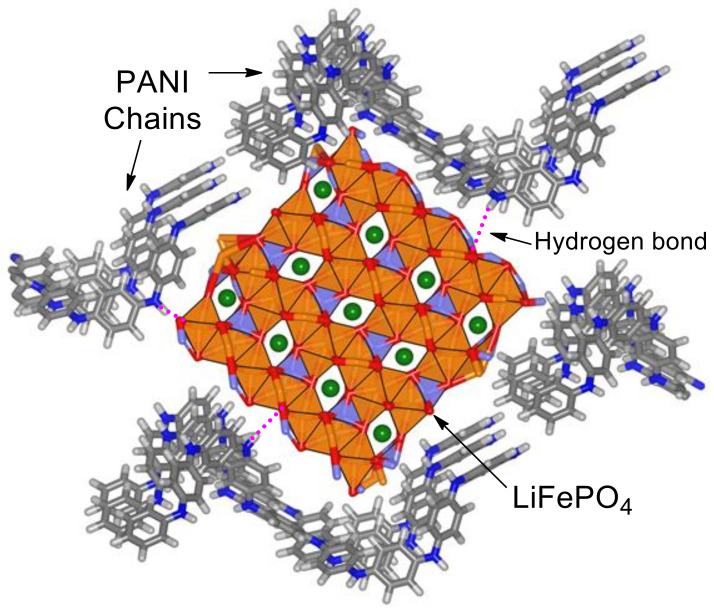
Qualitative structure for LiFePO_4_–PANI.

A qualitative structure of the LiFePO_4_–PANI is shown in Figure 21. The surface LiFePO_4_ particles are coated with PANI.

The electrochemical results of the LiFePO_4_–PANI reported in this study are similar to those of other works [12,40,41,42,43,44]. The specific capacity of LiFePO_4_–PANI depends of the methods of the synthesis, dopants in PANI and the electrochemical properties of the LiFePO_4_. All the works show an enhancement of the specific capacity at lower C-rates.

## 4. Conclusions

PANI was synthesized by chemical oxidation with good crystallinity, with primary particle size of 0.31 um and globular morphology forming agglomerates of 2.75 um. A LiFePO_4_–PANI composite was prepared via a thermal process, using LiFePO_4_, PANI and AcOLi. The morphologies of the LiFePO_4_–PANI and LiFePO_4_ particles are similar, but the PANI coating on the LiFePO_4_ particles gives a different surface structure in the composite. This is supported by elemental mapping, which shows a homogeneous distribution of carbon and sulfur in PANI on the surface of LiFePO_4_ particles.

The composite electrodes show good capacity, rate capability and cyclability. The composite containing 25 wt.% PANI shows better electrochemical performance compared with pure PANI and pure LiFePO_4_. The discharge capacity of PANI is 95 mAh g^−1^, that of LiFePO_4_ is 120 mAh g^−1^ and that of synthesized LiFePO_4_–PANI is 145 mAh g^−1^ at 0.1 C. At 2 C, the discharge capacity is 70 mAh g^−1^ for LiFePO_4_ and 100 mAh g^−1^ for LiFePO_4_–PANI. The PANI can be considered as a conductor for the electronic transfer between the LiFePO_4_ particles, but it also improves the contact between the electrolyte and LiFePO_4_ particles during the charge/discharge process. The PANI can also act as a host for Li^+^ ion insertion–extraction and gives an additional contribution to the capacity of the composite. PANI can also serve as a binder network between LiFePO_4_ particles and the current collector surface.

## Figures and Tables

**Table 1:** Data for impedance spectra at different cycling stages.

Material	R_1_ (Ohm)	R_ct_ (Ohm)
LiFePO_4_ as assembled (OCV)	14.13	178
LiFePO_4_–PANI as assembled (OCV)	3.58	61.1
PANI as assembled (OCV)	4.12	238

## Prime Novelty Statement

In this work, we developed a new route to synthesize a LiFePO_4_–PANI hybrid via thermal treatment, using lithium iron phosphate (LiFePO_4_), polyaniline (PANI) and lithium acetate (AcOLi) as starting materials. The synthesis process has a high yield (99.99%). The structural, morphological and compositional properties of the LiFePO_4_–PANI hybrid are characterized. Using the LiFePO_4_–PANI hybrid as electrode materials for lithium-ion battery application, good electrochemical performance was obtained, showing a discharge capability of 145 mAh g^−1^ at 0.1 C, 120 mAh g^−1^ at 1C and 100 mAh g^−1^ at 2 C, and cycling stability with 99.0% capacity retention at 0.1 C after 50 cycles. These initial research results are very interesting, and the technology developed in this work will provide a valuable approach to new potential cathode materials.

## Supplementary Materials

The following are available online at https://www.mdpi.com/1996-1944/13/12/2834/s1. Figure S1: Mechanism of aniline polymerization in the synthesis PANI. Figure S2: Morphology of PANI, a, upper) illustration of primary particles and agglomerates, b, lower) SEM images of the synthesized PANI agglomerates and enlargement showing the globular morphology. Figure S3: EDS spectrum of the PANI. Figure S4. FTIR spectra for PANI. Figure S5. Possible structure of the PANI and its interactions. Figure S6. Possible qualitative reactions of the decomposition of the PANI during the TG analysis. Figure S7. Possible qualitative reactions of the decomposition of the LiFePO_4_-PANI during the TG analysis. Figure S8. Electrochemical performance of PANI: cycling performance profiles. Figure S9. Electrochemical performance of PANI: galvanostatic discharge-charge profiles. Figure S10. Cell voltage as a function of time at different cycles obtained by charging and discharging at same rate for the LiFePO_4_-PANI. The data were obtained by charging and discharging at 0.1 C.
