# Supplementary material for: Synthesis and Characterization of LiFePO4–PANI Hybrid Material as Cathode for Lithium-Ion Batteries
Source: Materials (Basel). 2020 Jun 24;13(12):2834. doi: 10.3390/ma13122834 (PMC7344730; doi:10.3390/ma13122834)
Supplement: Supplementary file 1 [file materials-13-02834-s001.pdf]

materials

MDPI

Supplementary Materials:

Synthesis and Characterization of $LiFePO_4$–PANI Hybrid Material as Cathode for Lithium-Ion Batteries

Cesario Ajpi [1,2,*], Naviana Leiva [1], Max Vargas [1], Anders Lundblad [3], Göran Lindbergh [2] and Saul Cabrera [1,*]

1 Department of Inorganic Chemistry and Materials Science/Advanced Materials, IIQ Chemical Research Institute, UMSA Universidad Mayor de San Andres, La Paz, 303 Bolivia; cesario.ajpi@gmail.com (C.A.); nindeleivqmc@gmail.com (N.L.); vargmax@gmail.com (M.V.); saulcabreram@hotmail.com (S.C.)

2 Department of Chemical Engineering, Applied Electrochemistry, KTH Royal Institute of Technology, SE-10044 Stockholm, Sweden; gnli@kth.se

3 Division of Safety and Transport/Electronics, RISE, Research Institutes of, Sweden, SE-50462 Borås, Sweden; anders.lundblad@ri.se

* Correspondence: cesario.ajpi@gmail.com (C.A.); nindeleivqmc@gmail.com (N.L.)

Synthesis and Structural Characterization of PANI

Synthesis of PANI

The possible mechanism of the polymerization is shown in Figure.S1.

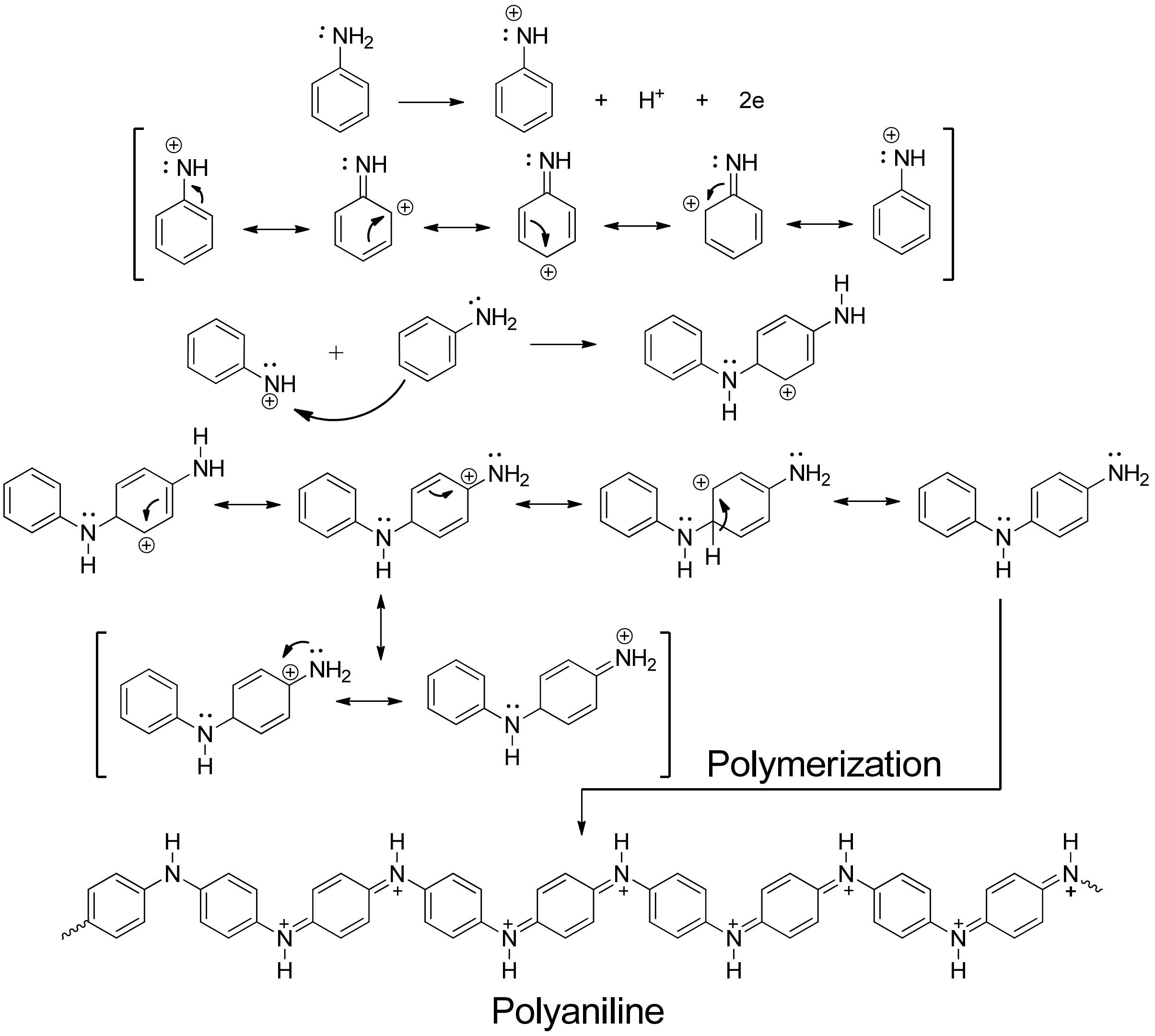

Figure S1. Mechanism of aniline polymerization in the synthesis PANI.

Figure S2 shows SEM images of PANI. The resulting powder of PANI synthetized by a self-assembly process has globular morphology with aglomerates of an average diameters of 2.75 μm, conformed by primary particles of an average diameter of 310 nm, The globular morphology, characteristic of PANI synthesis by chemical oxidation is shown in Figure S3a.

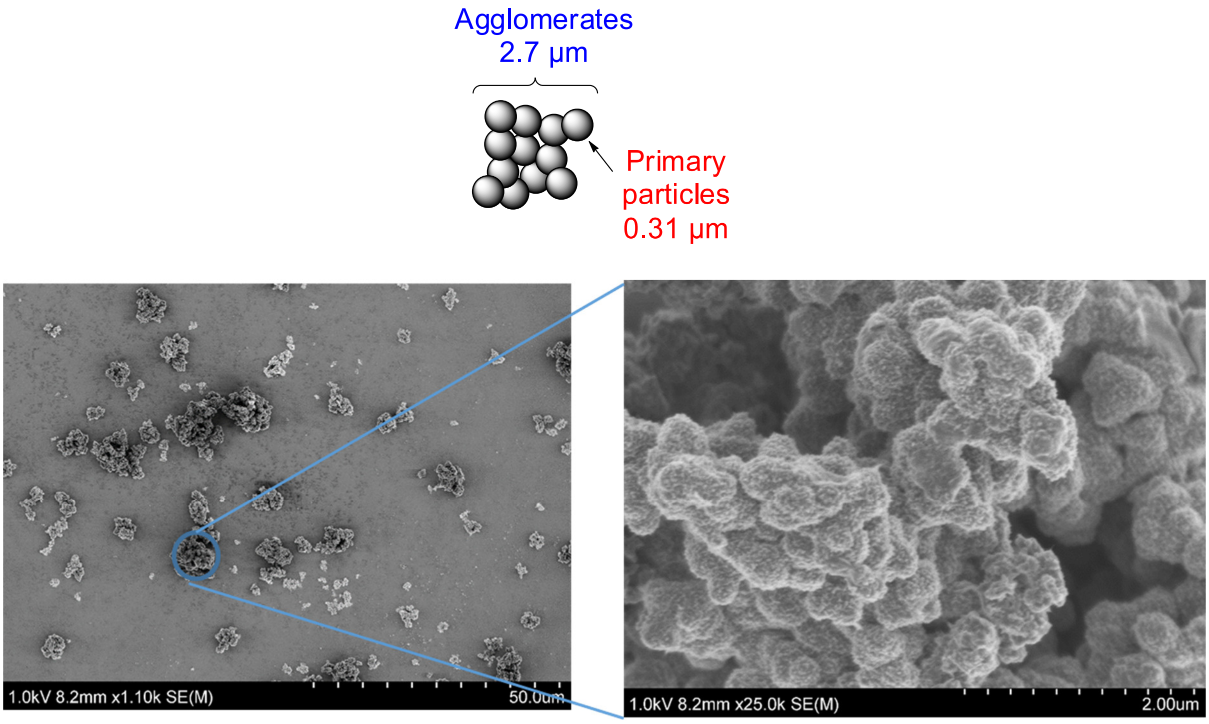

Figure S2. Morphology of PANI, a, upper) illustration of primary particles and agglomerates, b, lower) SEM images of the synthesized PANI agglomerates and enlargement showing the globular morphology.

The EDS depicts a characteristic composition of the PANI compound. The elemental composition is presented in Figure S3.

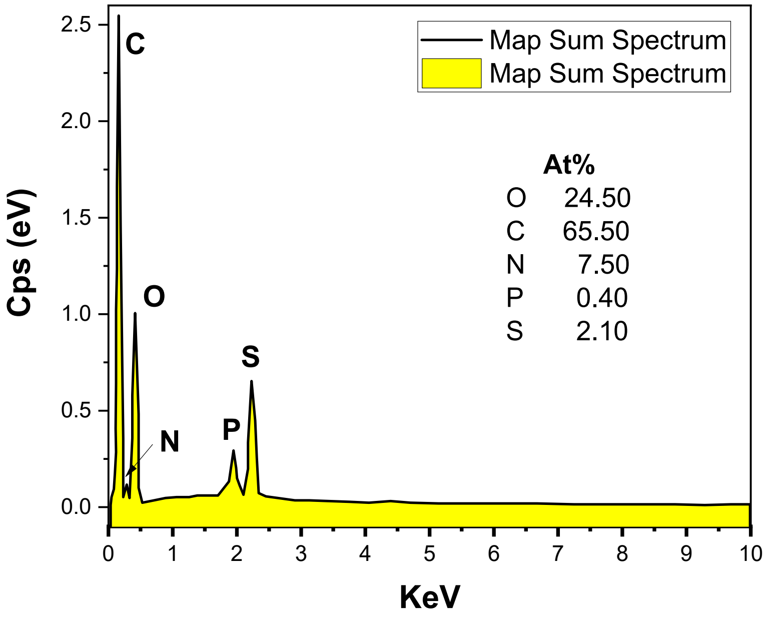

Figure S3. EDS spectrum of the PANI.

FT-Infrared spectroscopy

The peaks around 3230 cm^{-1} and 2464 cm^{-1}, correspond to stretching of O-H of the group HSO_4^- [1].

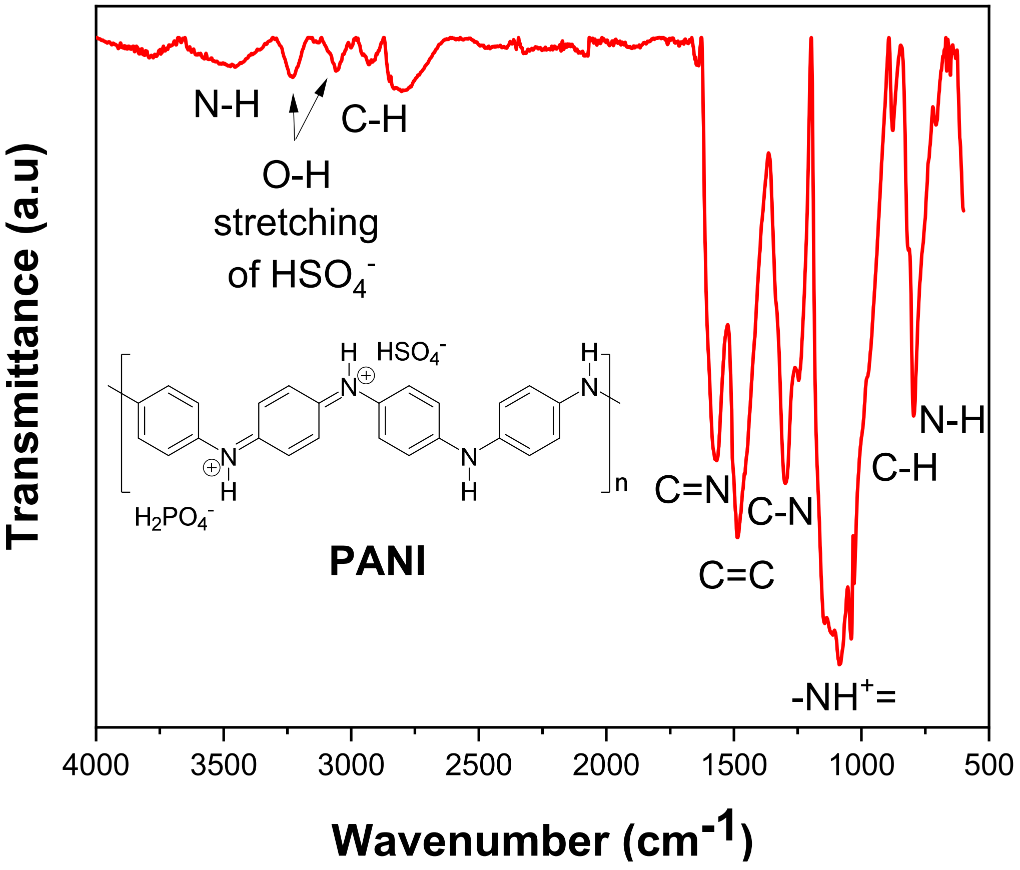

Figure S4. FTIR spectra for PANI.

A possible structure and interactions in the PANI is shown in Figure S6.

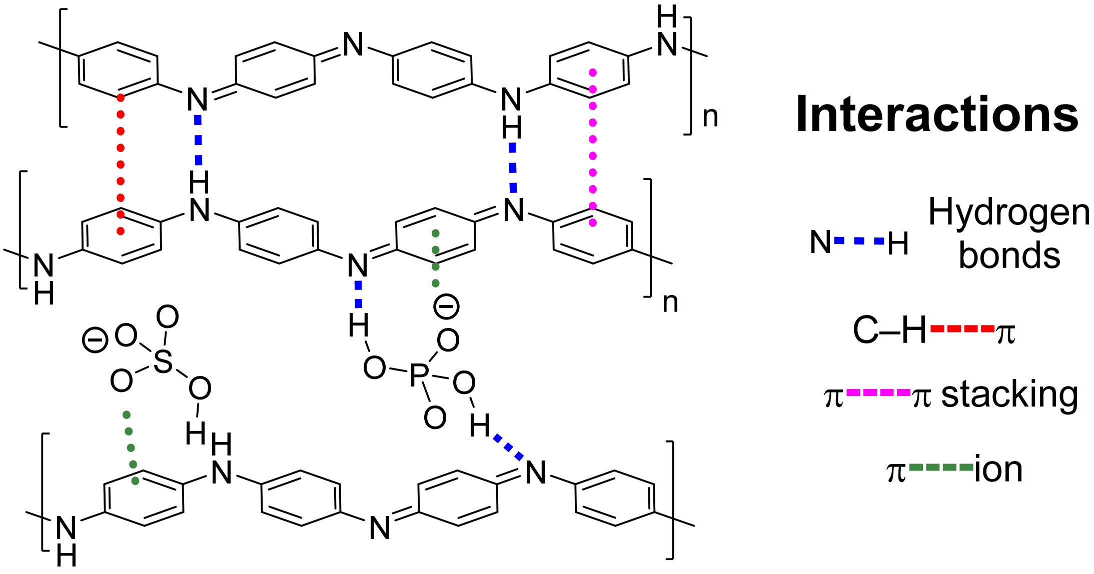

Figure S5. Possible structure of the PANI and its interactions.

The possible interactions present in the structure are hydrogen bonds, ion-ion, lone pair-π and ion-π. These interactions are weak except the hydrogen bond. The role of those interactions defines the crystallinity of the PANI and the packing of the chains in the structure.

Thermogravimetric analysis of PANI

Figure S6 shown the possible decomposition reactions to explain the weight loss in each range of temperature.

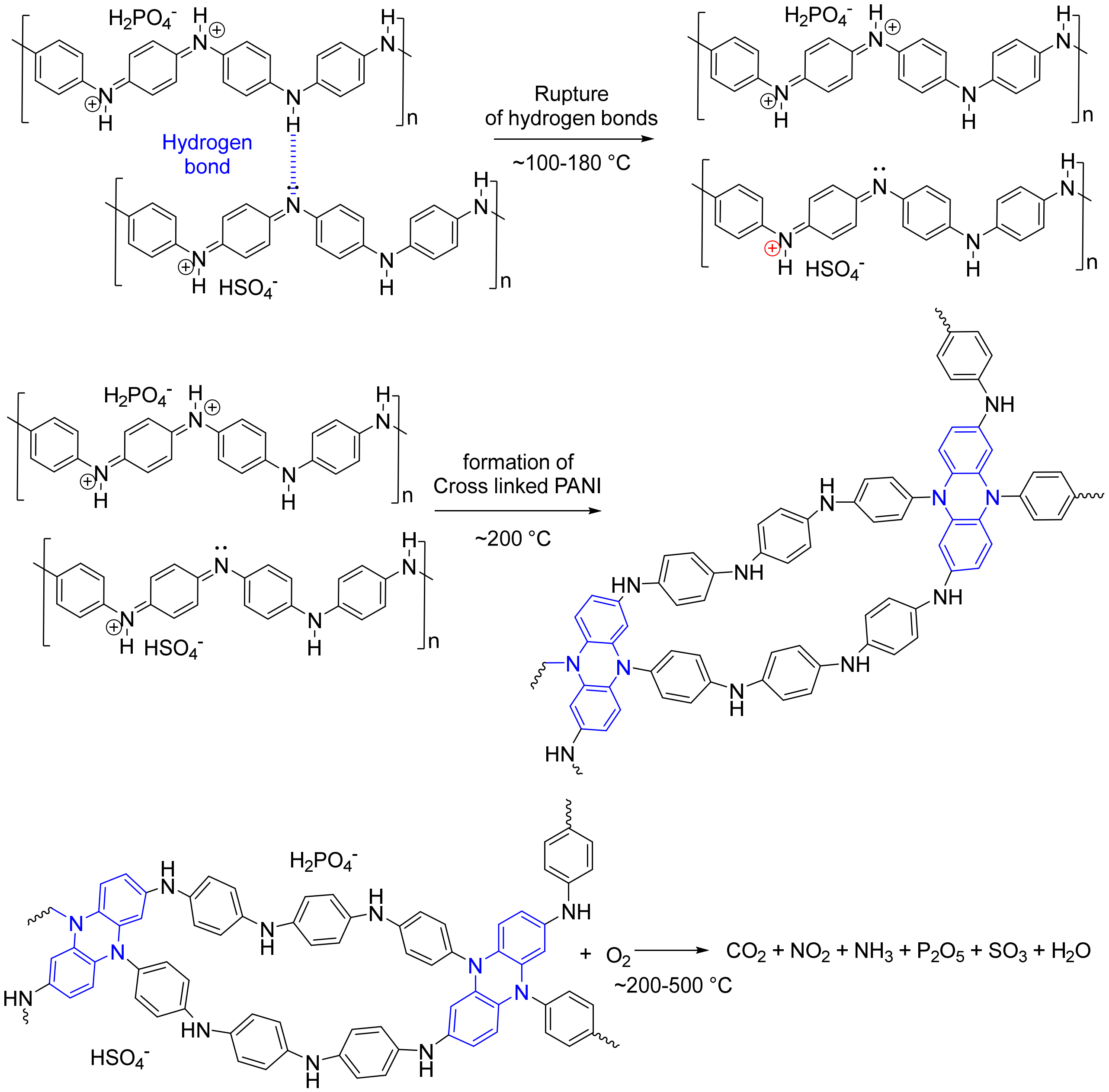

Figure S6. Possible qualitative reactions of the decomposition of the PANI during the TG analysis.

Figure S7 shown the possible decomposition reactions to explain the weight loss in each range of temperature.

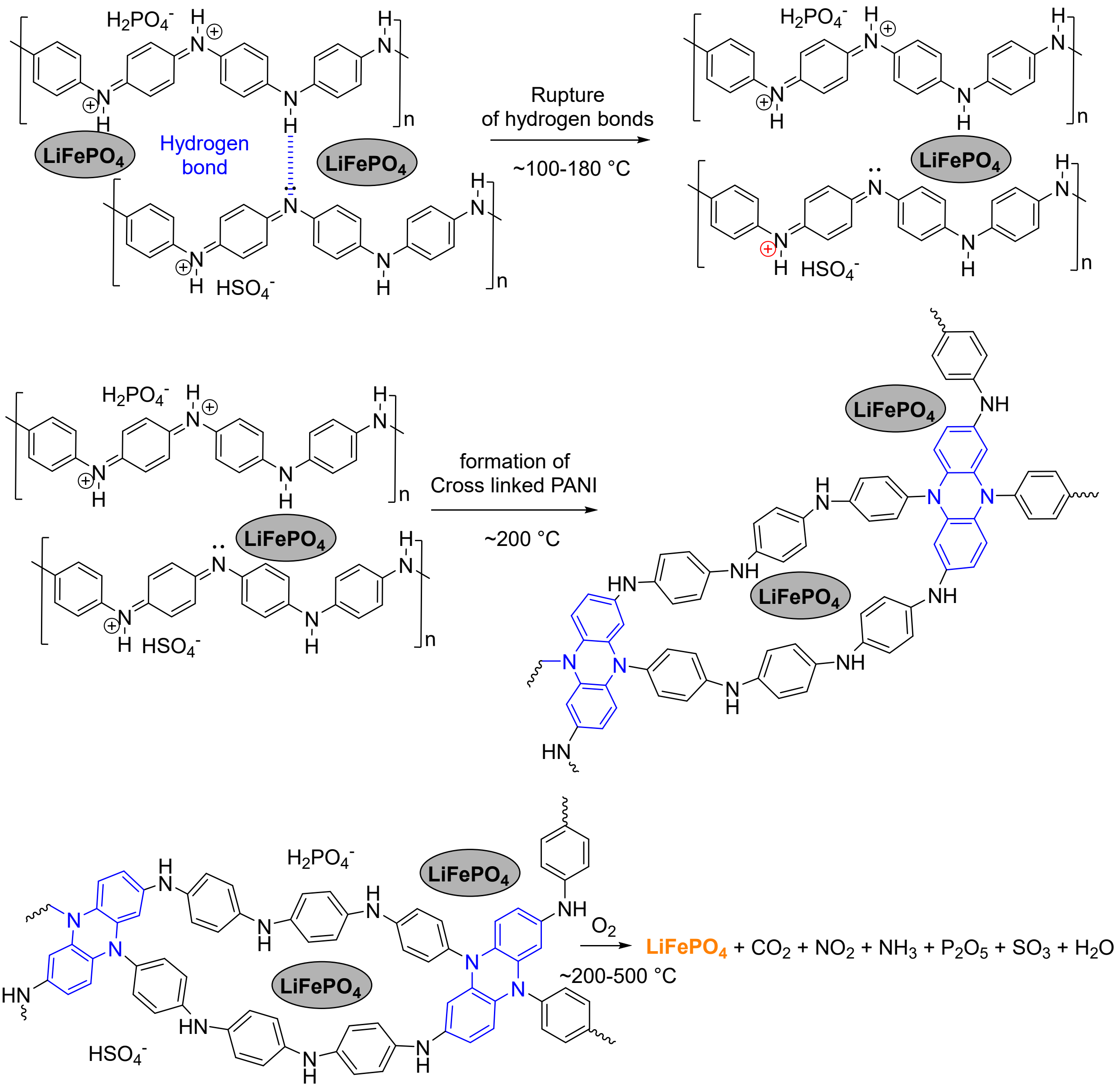

LiFePO4

$$4LiFePO_4 + O_2 \xrightarrow{\sim 500\text{-}700\ ^\circ C} 2Li_2O + 4FePO_4$$

$$2Li_2O + 4FePO_4 \longrightarrow 4LiFeO_2 + 2P_2O_5$$

$$4LiFeO_2 + 4O_2 \longrightarrow 2Li_2O + 2Fe_2O_3$$

Figure S7. Possible qualitative reactions of the decomposition of the LiFePO4-PANI during the TG analysis.

Electrochemical characterization of PANI

Charge-discharge

The PANI composite was electrochemically tested in a pouch test cell by charging and discharging at room temperature over 10 cycles at a rate 0.1 C. Figure S8 show the charge-discharge capacity vs. cycle number and capacity vs. voltage. The PANI was tested in the range of 2.5–3.9 V. Figure S9 shows the charge and discharge profiles. The PANI shows a small capacity of 95 mAh g^{-1}.

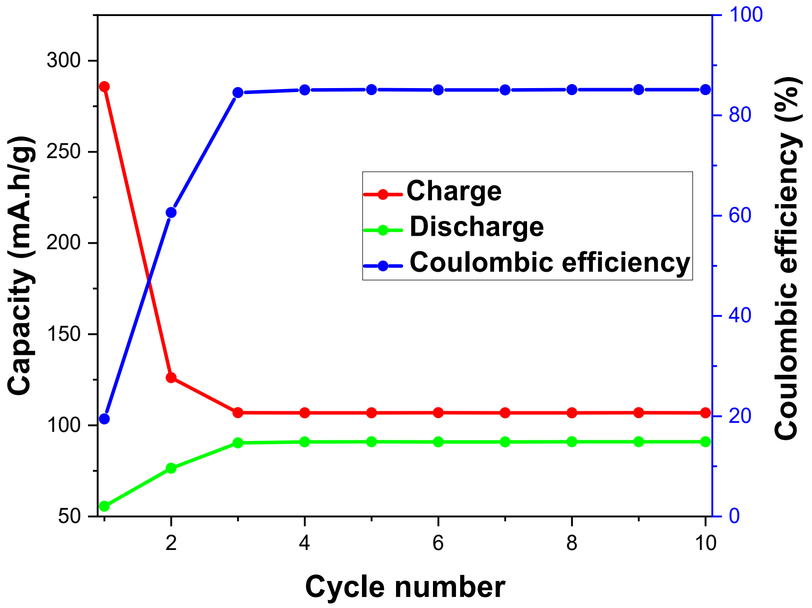

Figure S8. Electrochemical performance of PANI: cycling performance profiles.

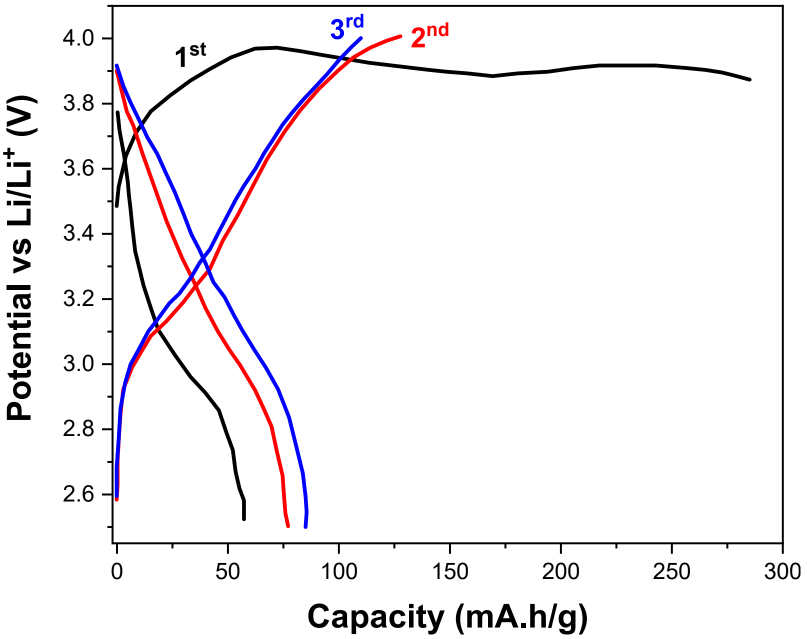

Figure S9. Electrochemical performance of PANI: galvanostatic discharge–charge profiles.

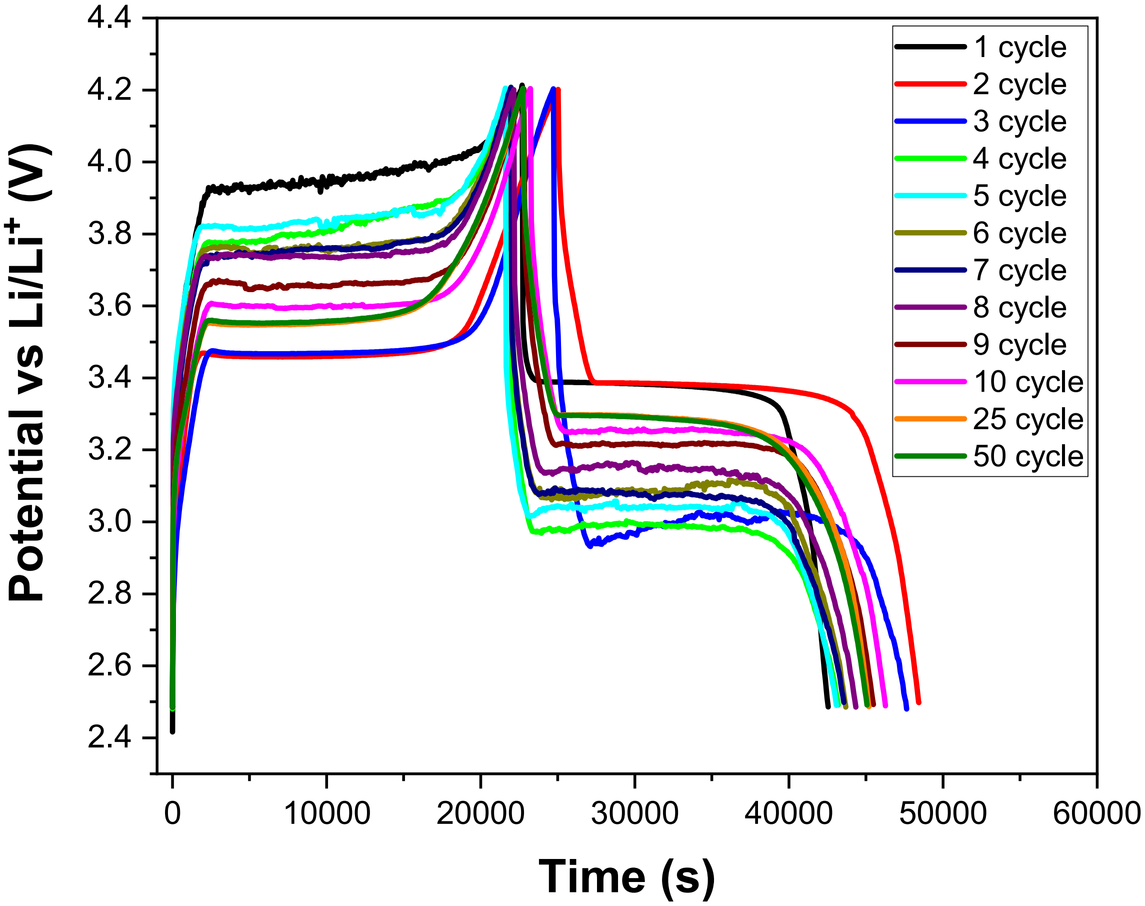

Figure S10. Cell voltage as a function of time at different cycles obtained by charging and discharging at same rate for the $LiFePO_4$-PANI. The data were obtained by charging and discharging at 0.1 C.

References

1. Periasamy A, Muruganand S, Palaniswamy M. Vibrational studies of Na_2SO_4, K_2SO_4, $NaHSO_4$ and $KHSO_4$ crystals. *Rasayan J. Chem*. 2009, *2*, 981–989.
